# The molecular and clinical role of Tensin 1/2/3 in cancer

**DOI:** 10.1111/jcmm.17714

**Published:** 2023-06-09

**Authors:** Laurensius Mainsiouw, Matthew Edward Ryan, Sassan Hafizi, Jason C. Fleming

**Affiliations:** ^1^ School of Medicine University of Liverpool Liverpool UK; ^2^ Department of Molecular and Clinical Cancer Medicine, Faculty of Health and Life Sciences University of Liverpool Liverpool UK; ^3^ Liverpool Head and Neck Centre University of Liverpool Liverpool UK; ^4^ School of Pharmacy and Biomedical Sciences University of Portsmouth Portsmouth UK

**Keywords:** cancer, neoplasm, Signalling, Tensin, *TNS1*, *TNS2*, *TNS3*

## Abstract

Tensin 1 was originally described as a focal adhesion adaptor protein, playing a role in extracellular matrix and cytoskeletal interactions. Three other Tensin proteins were subsequently discovered, and the family was grouped as Tensin. It is now recognized that these proteins interact with multiple cell signalling cascades that are implicated in tumorigenesis. To understand the role of Tensin 1–3 in neoplasia, current molecular evidence is categorized by the hallmarks of cancer model. Additionally, clinical data involving Tensin 1–3 are reviewed to investigate the correlation between cellular effects and clinical phenotype. Tensin proteins commonly interact with the tumour suppressor, DLC1. The ability of Tensin to promote tumour progression is directly correlated with DLC1 expression. Members of the Tensin family appear to have tumour subtype‐dependent effects on oncogenesis; despite numerous data evidencing a tumour suppressor role for Tensin 2, association of Tensins 1–3 with an oncogenic role notably in colorectal carcinoma and pancreatic ductal adenocarcinoma is of potential clinical relevance. The complex interplay between these focal adhesion adaptor proteins and signalling pathways are discussed to provide an up to date review of their role in cancer biology.

## 1 INTRODUCTION

**FIGURE 1:**
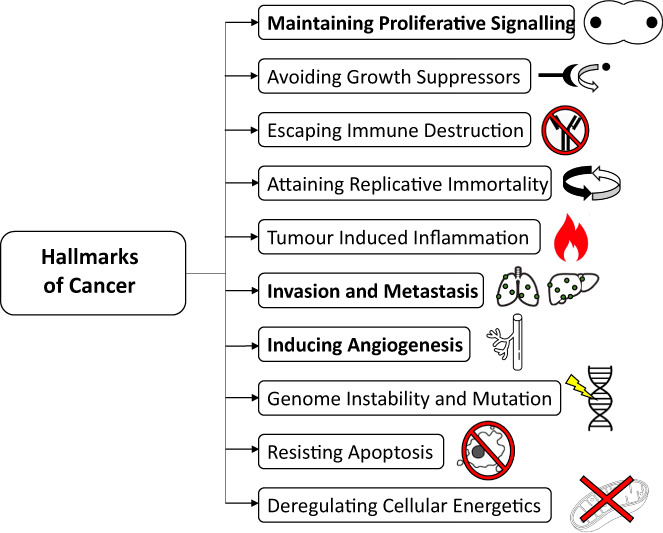
Hallmarks of cancer as adapted from Hanahan & Weinberg. The traits in bold will be the main focus of this review as the current evidence concerning Tensin 1, 2 and 3 is limited/non‐existent when discussing the other hallmarks.

Local and systemic processes defining cancer are summarized in the classic and modified ‘hallmarks of cancer’, providing a conceptual tool to visualize a multi‐pathway system (Figure 1).^1^ Over the previous two decades, a mechanistic link between the family of structural proteins known as Tensins and primary malignancies has been proposed.^2^, ^3^, ^4^, ^5^ The initial hypotheses on the four members of the Tensin family focussed on their role as either structural support or focal adhesion adaptor proteins, providing a critical link between the extracellular matrix (ECM) and intracellular cytoskeleton.^6^, ^7^ However, this was likely an oversimplification with more recent evidence suggesting additional signal transduction roles. Furthermore, the dysregulation of focal adhesion complexes is one recognized mechanism behind the ability of cancer cells to metastasise. A clear role or mechanism of action in oncogenesis for Tensins has not been elucidated, and findings appear tumour type‐specific.^2^ The growing body of evidence for a diagnostic, prognostic and potential therapeutic role for these proteins mandates a clearer understanding of their molecular biology and oncogenic function. Tensin 4 (CTEN) has received the most focus in the literature and was recently reviewed in Liao et al (2021).^8^ The remaining three Tensin family members share partial domains with Tensin 4, and we therefore hypothesize that they similarly have potentially important functions in cancer biology and are the focus of this review, using three established hallmarks to present current evidence.

## 2 MOLECULAR STRUCTURE OF TENSIN PROTEINS

**FIGURE 2:**
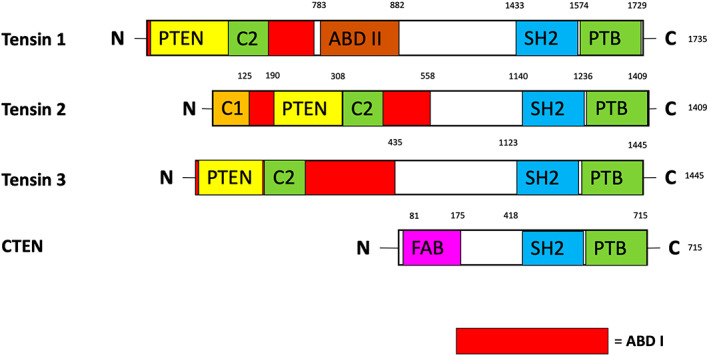
Schematic diagram of the four Tensin proteins, numbers denote amino acid number. The red region on Tensin proteins 1–3 indicates the first Actin binding domain (ABD I). Domains highlighted in boxes along sequence. PTEN (Phosphatase and Tensin Homologue), SH2 (Src Homology 2), PTB (Phosphotyrosine Binding Domain), C1 (Protein kinase C conserved region 1). Interestingly C1, PTEN and C2 domains commonly overlap the ABD I domain. Finally, CTEN is unique as it appears to be a truncated form of the previous three proteins. It also differs with a N‐terminus FAB (Focal Adhesion Binding) domain instead of the typical ABD I. All of these domains are considered important as they are sites of protein interaction.

This four‐protein family comprising Tensin 1 (*TNS1*), 2 (*TNS2/TENC1/C1‐TEN*), 3 (*TNS3*) and CTEN (C‐Terminal Tensin‐like, *TNS4*) share a high degree of sequence similarity, specifically the C‐terminal region comprising Phosphotyrosine binding (PTB) and Src Homology 2 (SH2) domains. However, CTEN structure is comparatively truncated, losing the conserved actin binding domain found in the N‐terminus of Tensin 1–3 (Figure 2). This has functional consequences unique to these Tensin family members. Tensin 1–3 proteins share an N‐terminal actin binding domain (ABD) with sequence homology to PTEN phosphatase, and a middle linker region. However, there are some important structural differences between them; Tensin 1 contains a 2nd ABD in the middle linker region; Tensin 2 variably contains a protein kinase C conserved region 1 (C1) domain before the ABD (Figure 2).^6^, ^9^ C1 domains have been classically recognized as phorbol ester‐binding modules such as exists in protein kinase C.^10^ The C1 domain in Tensin 2 is atypical due to low amino acid sequence identity with classic C1 domains. This previously evidenced in our study of the individual domains of Tensin 2, which demonstrated that the C1 domain was notable for localizing to the nucleus both alone and as part of a Tensin 2 fragment.^9^ The functional implications this property of the C1 domain has on cells have not been comprehensively defined, including in tumours expressing this particular isoform of Tensin 2.^8^ Moreover, it does pose the question if and how do these unique structures impart different signal transduction functions for each protein, many of which are linked to the hallmarks of cancer.^1^ Furthermore, they provide potential binding sites for pharmacological targeting unique to each Tensin.

## 3 GENE REGULATION

Several studies have provided insights into regulation of expression of Tensins at various levels; much of this work has focused on CTEN but although not a primary focus of this review, it may lead to novel insights on regulation of other Tensin family members. Chen et al. identified a minimal 327 bp region within the human *CTEN* gene promoter that showed more pronounced activity in prostate cell lines vs other cell types.^11^ These findings could be relevant to the frequent observation of CTEN downregulation in prostate cancer in contrast to its upregulation in many other cancer types. The same group also reported that CTEN is a downstream target of ΔNp63α, a member of the p53 transcription factor family, which is also downregulated in prostate cancer.^12^ ΔNp63α was shown to directly interact with the *CTEN* promoter and induced its transcriptional activity. Also, ChIP‐seq analysis revealed *TNS1* to be a direct target gene of the mineralocorticoid receptor in murine kidney tubular epithelial cells.^13^

The epigenetic regulation of Tensin gene expression has also been investigated. We have reported a differential pattern of DNA methylation in the promoter of the *TNS3* gene occurring in human renal cell carcinoma tumours vs normal tissue,^14^ which is linked to the downregulation of Tensin 3 expression in that cancer.^15^ In addition, *TNS3* was identified as a target gene for MLL3, a histone H3K4 methyltransferase that is frequently mutated in cancer.^16^ This therefore revealed Tensin 3, through its anti‐migratory capacity, to be part of the mechanism for MLL3 in suppressing cancer. By contrast, another study showed that EGF stimulation of cells, through MAPK signalling, enhanced the levels of histone acetylation within the CTEN gene promoter, thus upregulating CTEN gene expression.^17^ Furthermore, at the post‐translational level, both caspase and calpain families of cysteine proteases have been shown to proteolytically degrade Tensins, which can therefore affect the focal adhesion complex and thus negatively regulate Tensin‐mediated signalling in general.^18^, ^19^

## 4 COMMON INTERACTION WITH DELETED IN LIVER CANCER (DLC) PROTEIN

**FIGURE 3:**
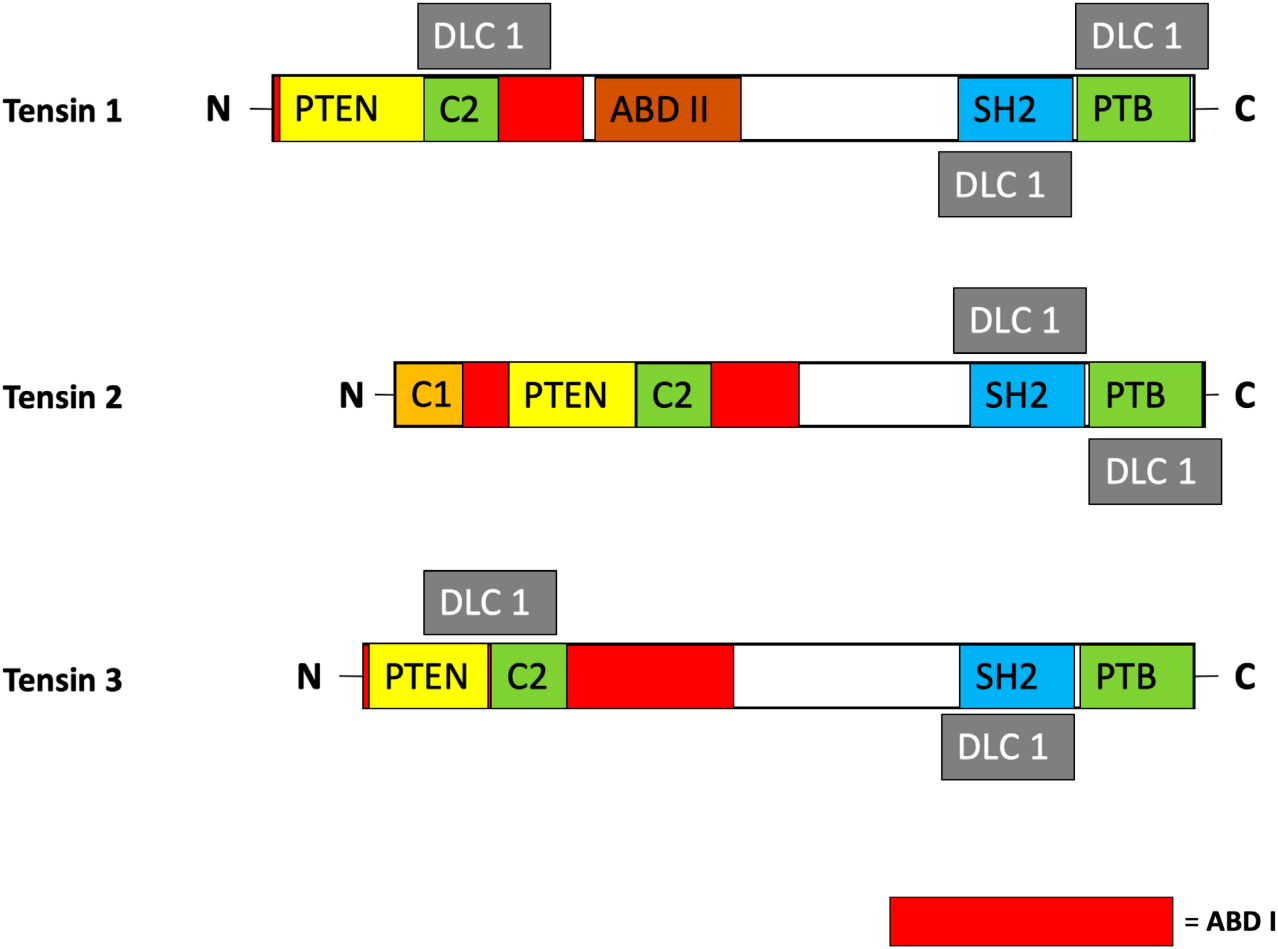
Schematic diagram of the three Tensins highlighting relevant domains that are involved in binding with DLC1. It is important to note that this is a simplification as the proteins are labelled in a two‐dimensional format to more easily describe the possible domain binding partners of DLC1. The C terminal SH2/PTB domains are a common binding site for DLC1 between the proteins. However, the N‐terminal C2/ABD I domains present in both Tensins 1 and 3 also interact with DLC1.

**FIGURE 4:**
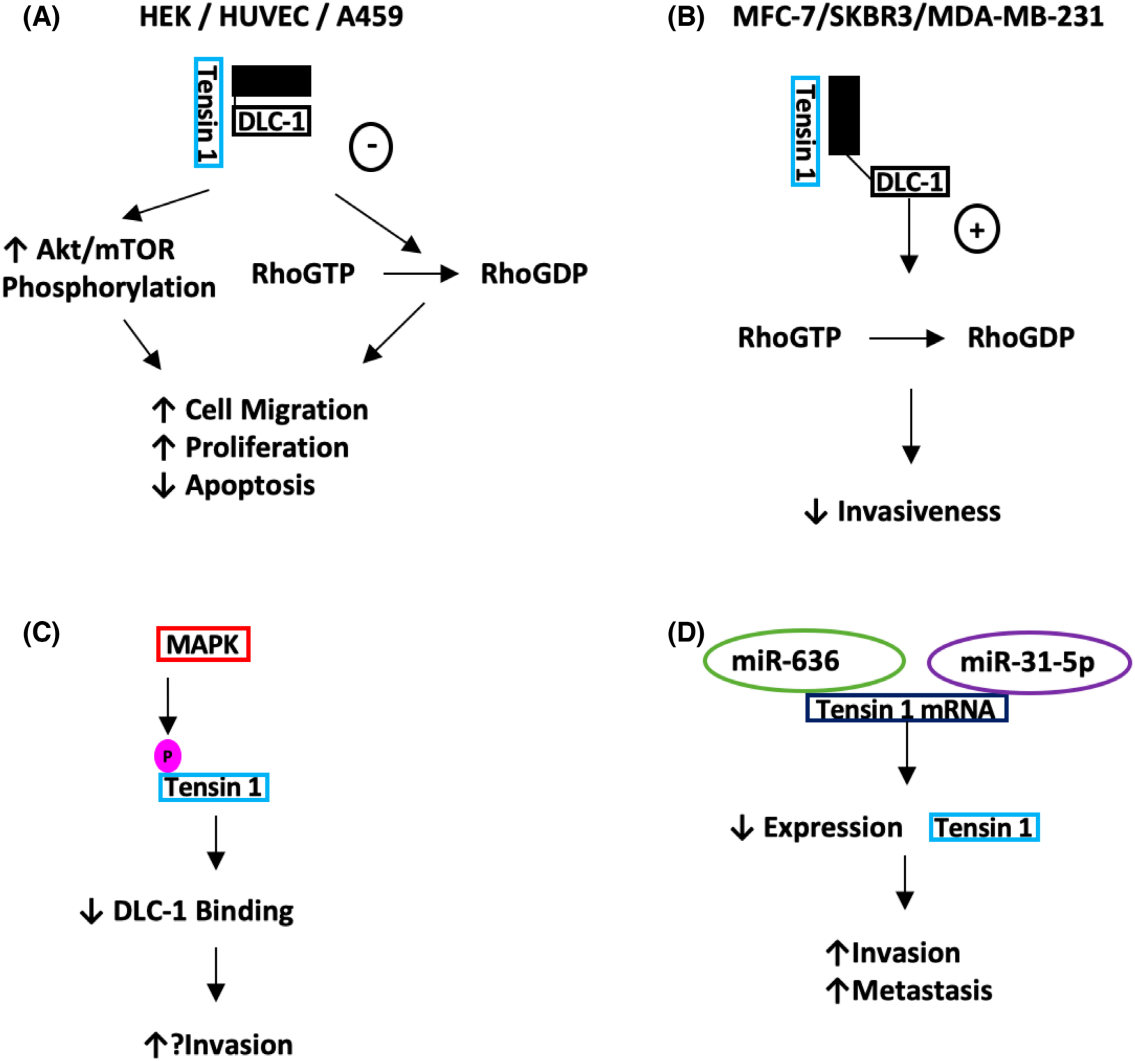
Simplified schematic overview of molecular mechanisms of Tensin 1. The role it plays in tumorigenesis is not clear. (A). Various studies have demonstrated that Tensin 1 promotes neoplastic traits by inhibiting RhoGTPase DLC1. (B) However, others have highlighted its role in promoting RhoGTPase, resulting in a reduction in invasiveness in breast cancer cell lines. (C) Phosphorylation experiments involving MAPK and Tensin 1 demonstrated decreased affinity for DLC 1 and potentially a decrease invasiveness of cells. (D). Studies in prostate and bladder cancer demonstrated role of Micro RNA 636 + 31‐5p, respectively, in decreasing expression of Tensin 1. This resulted in an increase in invasiveness and metastatic spread. Potentially using the same mechanism as shown in (B).

**FIGURE 5:**
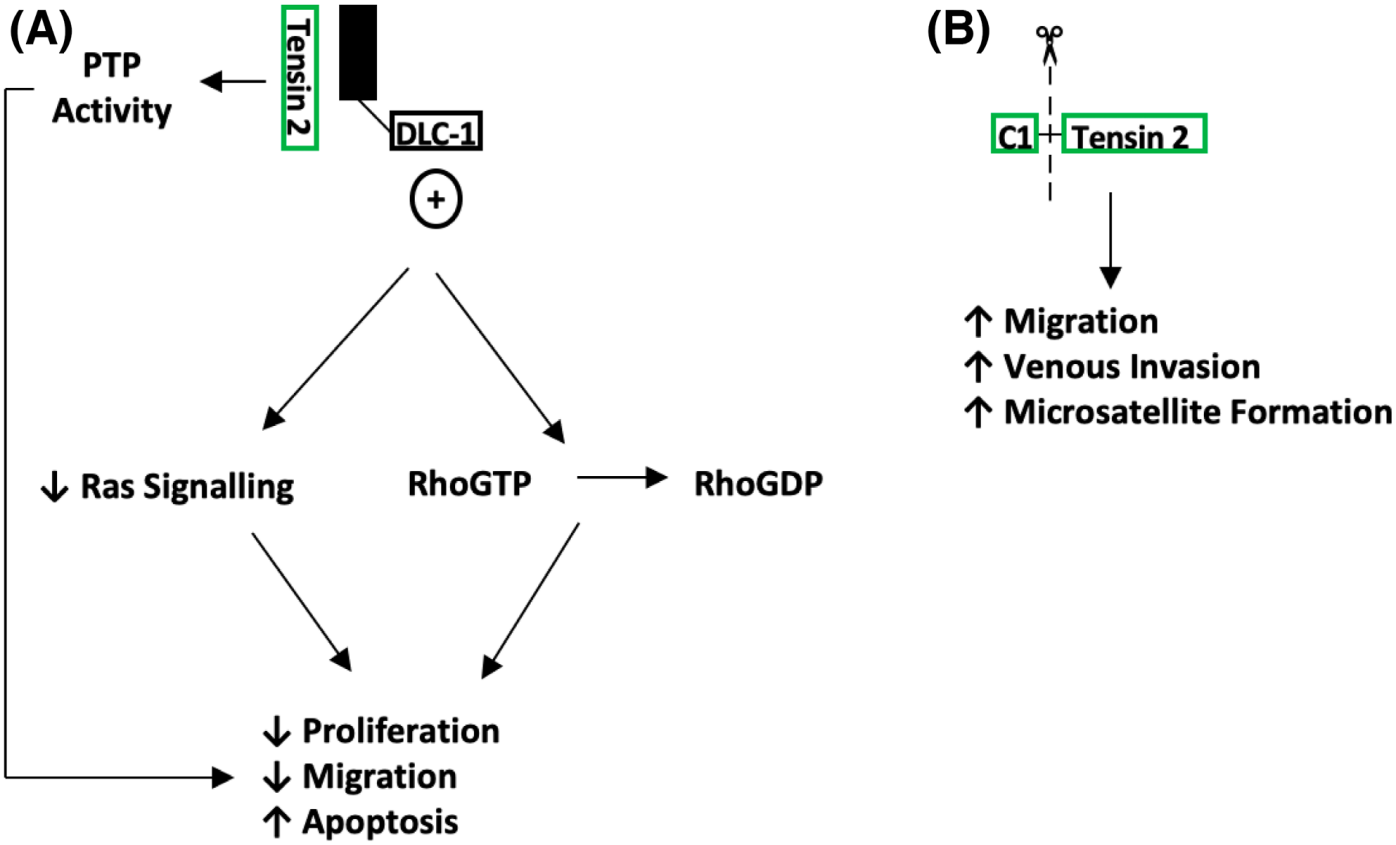
Molecular overview of Tensin 2. (A). Majority of evidence points towards Tensin 2 as a tumour suppressor by promoting DLC1 RhoGTPase activity, but also through other signalling pathways for example, PTP (protein/lipid phosphatase). (B) Other studies have demonstrated a C1 domain deficient variant of Tensin 2 potential roles in promoting tumour invasion and metastasis.

**FIGURE 6:**
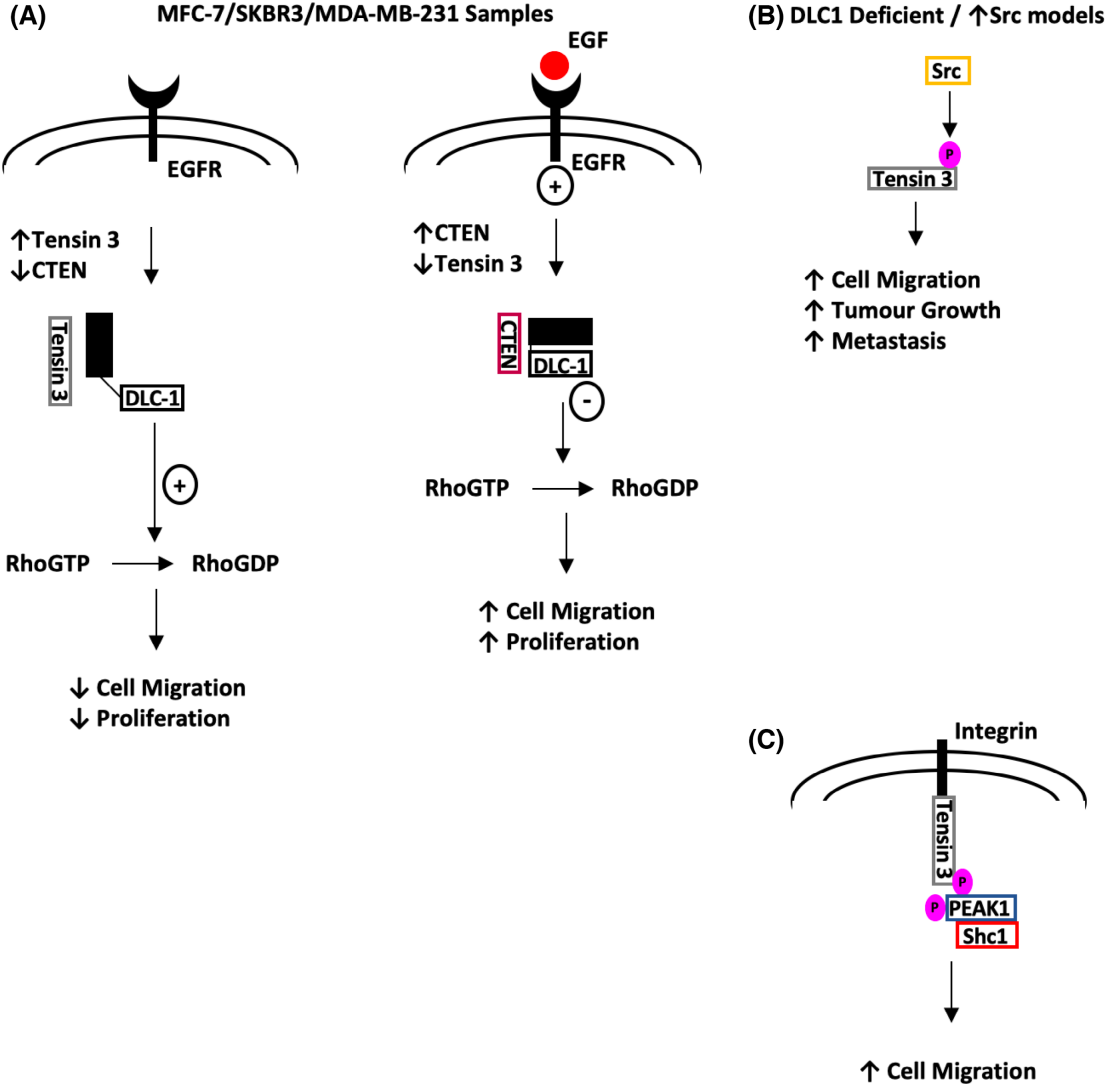
(A). Tensin 3 has been shown to act as a molecular switch with CTEN in mammary cell lines. If Tensin 3 is activated, it acts via DLC1 to promote anti‐tumour properties. (B) A subset of cancer cells were found with upregulated Src activity or are DLC1 deficient. In these cases, Tensin 3 is found to potentially exhibit pro tumour activity. (C) Association between a focal adhesion protein PEAK1 and Tensin 3 found that if both proteins are phosphorylated they form a complex with the intracellular component of specific integrins and another protein Shc1, which itself is involved in EGFR signalling. It was observed that these interactions lead to increased cell migration.

Interest in the role Tensin proteins play in tumorigenesis developed when Tensin 2 was found to interact with a protein commonly lost in hepatocellular carcinoma, deleted in liver cancer (DLC), or more specifically its three variants DLC 1,2,3.^20^, ^21^ As a RhoGTP activating protein (RhoGAP), DLC results in negative regulation of the *Rho* small GTPases family, causing a molecular switch to inactive RhoA GDP (Figures 4, 5, 6). This loss of active RhoA GTP may result in suppression of proliferation and cell migration via deactivation of the Rho‐associated protein kinase (ROCK) signalling pathway.^22^, ^23^ Tensins 1–3 appear to each have a different allosteric effect on DLC1 as discussed later, and different domains within Tensins 1–3 have been postulated to be the site of interaction (Figures 2, 3).^23^, ^24^ Therefore, dissection of this pathway is important in understanding their potential impact on oncogenic transduction pathways in multiple tumour types.

## 5 SUSTAINING PROLIFERATIVE SIGNALLING

### 5.1 Tensin 1

It was initially shown that the Tensin 1 SH2/PTB C‐terminal domains form a binding region with DLC1 which inhibits its RhoGAP function (Figure 2).^23^, ^25^ This was further reinforced when Shih et al. (2015) conducted studies involving ectopic expression of Tensin 1 and DLC1 in human embryonic kidney (HEK) cells. Tensin 1 was shown to increase the concentration of RhoGTP, thus reflecting the inhibition of the DLC1 RhoGAP activity. Furthermore, in endothelial cells from *TNS1* knockout mice or human umbilical vascular endothelial cells (HUVEC) with *TNS1* knocked down by siRNA, the authors reported reduced RhoGTP levels, which were rescued to normal levels upon silencing of DLC1. It was also demonstrated that reduced *TNS1* levels caused diminished endothelial cell proliferation, migration and tube formation in vitro (Figure 4).^26^

The Tensin 1 – DLC1 axis was further elaborated by Hall et al. (2010), demonstrating that MAPK phosphorylation in the central region of the protein inhibits DLC1 binding (Figure 4),^1^, ^27^ and therefore potentially enhancing active Rho‐GTP levels. It is likely that this feedback loop is more complex, exemplified by the differing functional effects of Tensin 1 levels in different tissue types, as discussed later.

Tensin 1 has also been implicated in another important pathway, mTOR. Duan et al. (2021) demonstrated that *TNS1* transfection in non‐small cell lung cancer (NSCLC) cells caused increased mTOR/Akt/RhoA activity, which was associated with increased proliferation, cell motility and decreased apoptosis (Figure 4). Additionally, they showed higher *TNS1* expression in clinical NSCLC tissues than in adjacent normal lung tissues, which were correlated with poorer 5‐year survival.^28^

### 5.2 Tensin 2

As the first member of the Tensin family studied, Yam et al. (2006), Kawai et al. (2010) and Chen et al. (2012) demonstrated binding between DLC1 and the Tensin 2 PTB domain (Figure 2).^20^, ^29^, ^30^ This was further corroborated by Chan et al. (2009), who demonstrated absence of DLC1 binding in Tensin 2 lacking the PTB domain.^5^ Re‐introduction of Tensin 2 into hepatocellular carcinoma (HCC) Tensin 2‐deficient cells, decreased proliferation and increased apoptosis.^31^ Furthermore, Ras and Akt signalling, overactivated in many cancers, was also revealed to be decreased with co‐expression of DLC1 and Tensin 2.^1^, ^32^ Hong et al used a mouse xenograft system with *TNS2* knockout HeLa cells, observing increased tumour growth relative to controls,^4^ supporting the previous in vitro findings of a tumour suppressor role for Tensin 2 (Figure 5). This is further corroborated through reduced Tensin 2 expression in multiple cancer cell lines and clinically when comparing primary tumour samples vs healthy tissue.^4^, ^32^, ^33^ Furthermore, while Tensins 1–3 all possess a PTEN‐like protein tyrosine phosphatase (PTP) and C2 domain within their N‐terminal ABD regions, Tensin 2 appears unique in possessing functional phosphatase activity.^32^ This has been demonstrated against tyrosine phosphorylated protein substrates and evidence has highlighted it has PTEN‐like lipid phosphatase activity. Both of these functions have been shown to suppress Akt signalling.^34^, ^35^

### 5.3 Tensin 3

Katz et al. (2007) published seminal work which demonstrated a reciprocal relationship between Tensin 3 and CTEN in mammary cells, regulated by epidermal growth factor receptor (EGFR). CTEN displaces Tensin 3 from integrin at focal adhesions when EGFR is activated, implicating a direct relationship between a common oncogenic signalling pathway and Tensin family expression. Furthermore, increased mammary cell migration was observed when CTEN was upregulated and Tensin 3 abrogated (Figure 6).^2^ Although the mechanism of this relationship was hypothesized to be related to a structural linkage between the ABD region of Tensin 3, the cytoskeleton and the ECM; Cao et al. (2012) proposed an additional signalling effect. The decrease in cell migration was potentially linked to Tensin 3 ABD releasing the autoinhibition of DLC1 (Figure 6).^36^ Interestingly, a recent study by Zuidema et al. demonstrated that Tensin 3 (through its SH2 domain) mediates an indirect interaction between integrins and the scaffolding protein PEAK1. This interaction regulates cell migration as well as also appearing to converge integrin and growth factor receptor signalling pathways (Figure 6).^2^, ^36^, ^37^

Various Tensin 3 studies have revealed Tensin 3 expression to be linked with a tumour suppressing effect. For example, higher *TNS3* mRNA expression has been correlated with a reduction in cell migration and invasion in malignant thyroid cell lines, and Tensin 3 downregulation has been detected in kidney tumours.^15^, ^33^, ^38^ However, these findings have not been replicated in other cancers; for example, the Tensin 3 – CTEN switch was reported to be absent in multiple colorectal cancer (CRC) cell lines.^19^ The authors found that activation of EGFR increased levels of both CTEN and Tensin 3, although there was no elaboration of functional effects. Furthermore, work conducted in NSCLC and melanoma in vitro, and breast cancer in vivo suggests pro‐oncogenic properties of Tensin 3.^39^ Qian et al. (2009) showed that increased levels of Src activity promote phosphorylation of the SH2 domain of Tensin 3 (Figure 2), enhancing cell migration, tumour growth and metastasis. One hypothesis is that the level of DLC1 expression in a tumour may impact on the functional role of Tensin 3.^39^ These data could also imply that the tumour promoting activities of Tensin 3 may also require a high level of Src activity within cells (Figure 6), or through an indirect effect. For example, recent evidence demonstrates that Src levels positively effect either focal adhesion formation or amoeboid type movement dependent on the level of cell adherence. Thus, promotion of cell motility mediated through Tensin 3 may also depend on a cell's physical state and adherence properties.^40^

## 6 ACTIVATING INVASION AND METASTASIS

### 6.1 Tensin 1

Treatment and prognosis of solid malignancies are often determined by the tumour ability to invade local structures and metastasise to distant locations. Cell invasion through the basement membrane into surrounding stroma is a fundamental first step, and epithelial‐mesenchymal transition (EMT) is recognized as a key pathological driver in this transformation. During EMT, fully differentiated epithelial cells activate genetic programs typically utilized in embryogenesis to invade, resist apoptosis and disseminate. Okayama et al. (2015) revealed that EMT models using transforming growth factor β increased the phosphorylation of Tensin 1.^41^ Shinde et al. (2020) demonstrated an alternative role of Tensin 1 in EMT in breast cancer, whereby Tensin 1 was required to stabilize extracellular vesicles containing transglutaminase 2 (TG2), promoting EMT in breast cancer cells. These results were replicated in fat pad mice xenograft models where the presence of both TG2 and Tensin 1 resulted in larger pulmonary nodules.^42^ Clinicopathological data also supported this hypothesis with a 37.6‐fold increase of phosphorylated Tensin 1 in poor versus good prognostic NSCLC cohorts. Indeed, overall high Tensin 1 expression correlated with significantly reduced recurrence‐free survival and overall survival.^41^, ^42^

Tensin 1 function does not appear consistent across tumour types but rather appears tumour type‐specific. For example, although *TNS1* overexpression in non‐cancer cells promoted their migration,^43^ *TNS1* expression was found to be absent in a panel of breast and prostate cancer cell lines. Zhan et al. (2016) demonstrated that downregulation of *TNS1* expression in various breast cancer cell lines by microRNA miR548j resulted in increased cellular invasiveness. The miR548j is thought to prevent *TNS1* mRNA translation through binding to the 3′ untranslated region. Furthermore, Tensin 1 protein levels were also negatively correlated with the invading breast cancer cell lines expressing miR548j, and reversed with the addition of a miR548j inhibitor.^44^ Furthermore, increased levels of RhoGTP were found in relation to miR548j expression, implying Tensin 1 potentially interacts with DLC1 to promote RhoGAP function (Figure 4). In addition, in large‐scale bioinformatic studies, Zhu et al. (2020) and Tang et al. (2020) found a negative correlation between *TNS1* expression and microRNAs miR‐636 and mir‐31‐5p, both of which were greater in their levels in metastatic prostate and bladder cancer, respectively, compared to non‐metastatic samples.^45^, ^46^ Furthermore, Zhu et al observed that increased *TNS1* expression was shown to provide benefit in terms of biochemical recurrence‐free survival and disease‐free survival in prostate cancer patients.^45^ Tang et al. reported that lower Tensin 1 levels were correlated with higher tumour and nodal stages of bladder cancer.^46^ The above findings imply that Tensin 1 may be protective against invasion and metastasis in breast, prostate and bladder primaries, and therefore that Tensin 1 loss is associated with cellular transformation during tumorigenesis.

### 6.2 Tensin 2

There is less evidence for the role of Tensin 2 related to cell invasion. Initially, it was shown that overexpression of *TNS2* in HEK293 cells saw a reduction in phosphorylation of Akt and GSK3 kinases, both of which are known to be involved in tumorigenesis. These molecular changes were also associated with increased apoptosis and decreased cell migration (Figure 5).^32^ Similar to the Tensin 2 proliferation data, it appears that Tensin 2 suppresses invasion and metastasis in tumour cells. However, Yam et al observed that a Tensin 2 variant lacking the C1 domain (Figure 2) has been linked to increased cell proliferation, migration and colony formation.^31^ Tumour growth was detected in a BEL7402 HCC cell line mouse orthotopic liver xenograft model, where the C1 deficient Tensin 2 variant was upregulated, highlighting that the same variant showed increased venous invasion and microsatellite formation in clinical HCC samples (Figure 5).^31^

### 6.3 Tensin 3

Previous data regarding the CTEN‐Tensin 3 switch and evidence gathered in HEK cells alluded to the potential role of Tensin 3 in tumour invasion and metastasis.^2^, ^15^, ^36^ Overexpression of *TNS3* in human kidney cells and one melanoma cell line has been shown to reduce cell migration in a fibronectin‐mediated haptotactic migration assay.^15^ In a breast cancer metastasis assay, Veß et al. (2017) utilized the breast cancer cell line MDA‐MB‐468 and identified that a specific subset of the cells did not adhere to the culture plates and were surviving in free suspension, with Tensin 3 mRNA and protein deficiency proposed as the cause. Furthermore, on re‐expression of *TNS3* in this model, there was a 20‐fold increase in adherent cells compared to controls.^48^ This demonstrated that Tensin 3 plays an anti‐migratory role in breast cancer cell lines, corroborating Katz et al. and Cao et al's previous work.^2^, ^36^, ^48^ Veß et al. also commented on a Tensin 3 ‐ CTEN switch present in their cell line, albeit without an apparent relationship to EGF signalling. However, levels of Src expression in the MDA‐MB‐468 cell line were not explored, which may have contributed to Tensin 3 acting in an anti‐tumorigenic manner.^48^

A study assessing epigenetic mechanisms identified an association with Tensin 3 and invasion in a metastatic breast cancer cell line; Shinchi et al. (2015) demonstrated that a reduction in a specific histone methylation site directly correlates to decreased *TNS3* mRNA and decreased invasiveness, but not proliferation, in MDA‐MB‐231 cells.^49^ This is contradictory to the previous example using a similar breast cancer cell line (MDA‐MB‐468).^48^ However, similarly to the Veß et al. study, Shinchi et al did not study DLC1 or Src activity in these cells.^48^, ^49^ It is therefore difficult to interpret and compare both studies, despite both cell lines being derived from a triple negative breast cancer phenotype. One hypothesis is that MDA‐MB‐468 could have an active DLC1 component and a relatively lower activity Src component. The reverse could be true in MDA‐MB‐231 cells, providing explanation as to why the former displays Tensin 3 as a tumour suppressor and the latter as pro‐oncogenic. It could also be plausible that the C1‐Tensin 3 isoform, which is similar to Tensin 2, could explain this alternative physiological effect. Nevertheless, however complex, both results do highlight a significant role for Tensin 3 in cancer cell invasion and metastasis.

## 7 ANGIOGENESIS

Cancer growth is often exponential and uncontrolled, requiring rapid neovascularization in order to supply its excessive metabolic demands. However, new vessel growth is disorganized and chaotic but vital as tumour growth typically surpasses territories supplied by existing vasculature. The clinical relevance of this hallmark is highlighted by bevacizumab therapy, a monoclonal antibody that targets vascular endothelial growth factor, thus inhibiting angiogenesis in multiple cancer primaries.^1^

The role Tensin proteins play in angiogenesis is poorly understood. A study attempting to identify key targets of phosphorylation coincidentally found that *TNS1* expression was upregulated in the blood vessels of testicular cancer samples. It was also observed that *TNS1* was upregulated in blood vessels in a wide panel of normal tissues including bladder, colon, kidney, prostate and testis.^50^ This clinical data have been supported by in vitro models using *TNS1* gene knockout in human umbilical vascular endothelial cells (HUVEC) which resulted in decreased proliferation, migration and tube formation.^26^

An investigation evaluating the gene expression in blood vessels bordering different grades of glioblastoma found a positive association with Tensin 2. It was observed that vessels surrounding grade four glioblastoma had statistically significantly lower *TNS2* gene expression compared to vessels surrounding grade two or control samples.^51^ Similar to the Kiflemariam S et al Tensin 1 study, there were insufficient data to understand how Tensin 2 influences angiogenesis or the potential role it plays in developed vessels associated with higher grade glioblastoma. However, taken in context with previous studies, it does appear to strengthen the argument regarding the tumour suppressive nature of Tensin 2.

## 8 CLINICAL CORRELATION

### 8.1 Tensin 1

There are mixed data regarding the clinical impact of Tensin 1 expression. As previously discussed, data from Zhu et al. and Tang et al. suggested that *TNS1* has a protective effect in prostate and bladder cancer (Table.1).^45^, ^46^ Both studies used a robust combination of large sample bioinformatic (200+ patients' genomes) and smaller sample histopathological datasets (30–45 patient samples). However, in alternate bioinformatic and biochemical studies conducted by Zhang et al and Chang et al, it was suggested that *TNS1* was a strong risk predictor in muscle invasive bladder cancer and breast cancer, respectively. Zhang et al. (2019) demonstrated that *TNS1* was more prevalent in the genomes of deceased bladder cancer patients in comparison with living patients. Additionally, expression was negatively correlated with overall survival.^52^ Chang et al. (2020) observed the effects of long non‐coding RNA MaTAR 25 in mammary carcinoma cell lines, one of its downstream effects being increased expression of *TNS1*, which resulted in metastasis and poor patient prognosis (Table.1).^53^

**TABLE. 1:** Clinical data concerning Tensin1/TNS1 and effect on various cancer primaries. Colorectal Cancer (CRC), Renal Cell Carcinoma (RCC).

Tensin 1/TNS1			
Primary	Effect on Cancer	Findings	Reference
RCC	↓Risk	↓mRNA Levels in RCC Negative Correlation with RCC Grade	Martuszewska et al.^27^
Prostate	↓Risk	↑Expression = ↑biochemical free survival and ↑disease survival.	Zhu et al.^36^
Bladder	↓Risk	↓Expression = ↑Metastases and ↓Prognosis.	Tang et al.^37^
Breast	↑Risk	↓Expression = ↑higher tumour and ↑nodal stage disease.	Chang et al.^44^
Bladder	↑Risk	↑Expression in deceased patients genomes and ↓overall survival.	Zhang et al.^43^
CRC	↑Risk	TNS1 gene amplified in CRC samples.	Burghel et al.^45^
CRC	↑Risk	↑Expression = ↓overall survival and ↓disease‐free survival	Mi et al.^46^
CRC	↑Risk	↑Expression = ↓overall survival and ↓disease‐free survival	Zhang et al.^47^
CRC	↑Risk	↑Expression in advanced stromal tissues = ↑Disease stage + ↓overall survival	Liu et al.^48^

Although the clinical implications of *TNS1* expression may be unclear at present in bladder, breast and prostate primaries,^45^, ^46^, ^52^, ^53^ in colorectal cancer (CRC) the effect of Tensin 1 expression appears more consistent, with four studies supporting an oncogenic role. Burghel et al. (2013) conducted a study on the genomes of 53 patients with microsatellite stable CRC. Regions of small chromosomal changes termed focal minimal common regions (FMCR), allow identification of specific gene variations that may drive cancer. It was demonstrated that *TNS1* is commonly found to be amplified in their CRC samples, suggesting an oncogenic effect (Table.1).^54^ This is further corroborated by Mi et al. (2020) and Zhang et al. (2019), where both groups found that increased expression of *TNS1* is associated with a poorer overall survival and a reduced disease‐free survival in CRC patients.^55^, ^56^ Mi et al. utilized four separate databases containing CRC patient genomic information. Zhang et al. assessed microRNA expression in lymph node metastasis and found a bioinformatic link with *TNS1* (Table.1). Furthermore, clinical data suggest that *TNS1* has a role in the stroma surrounding CRC. Liu et al. (2020) found stroma surrounding the CRC primary was more advanced in higher stage disease.^57^ Liu et al. additionally observed *TNS1* levels were increased in advanced stromal tissues while simultaneously being associated with worse overall survival and a higher stage cancer (Table.1).^57^ Despite this strong clinical/bioinformatic evidence, the precise biological mechanism of oncogenesis remains elusive but a focus on in vitro*/*in vivo work in CRC would appear to be a selective tumour approach with strong clinical potential.

### 8.2 Tensin 2

The role of Tensin 2 as a tumour suppressor appears well supported by pre‐clinical data, a trend mirrored in clinical studies, with almost universal agreement. Yang et al and Hong et al used bioinformatic approaches interrogating various online cancer genome databases. They found that *TNS2* acts as a tumour suppressor in multiple tissue types. This was achieved by comparing *TNS2* expression in normal tissue vs cancer tissue. *TNS2* expression was identified as being significantly lower in cancer tissues of head and neck squamous cell, oesophageal squamous cell, breast, NSCLC, HCC, ovarian and CRC datasets (Table.2).^4^, ^58^ Furthermore, lower *TNS2* expression was correlated with poor relapse free survival probabilities in one lung and one breast cancer dataset (Table.2).^4^

**TABLE. 2:** Clinical data concerning Tensin2/TNS2/TENC1/C1‐TEN and effect on various cancer primaries. Non‐small cell lung cancer (NSCLC), Hepatocellular Carcinoma (HCC), Colorectal Cancer (CRC), Pancreatic Ductal Adenocarcinoma (PDAC), Renal Cell Carcinoma (RCC), Gastrointestinal Stromal Tumour (GIST).

Tensin 2/TNS2/TENC1/C1‐TEN			
Primary	Effect on Cancer	Findings	Reference
Head & Neck Squamous Cell	↓Risk	↓Expression in cancer in 22 paired sample dataset.	Hong et al.^4^
Oesophageal Squamous Cell	↓Risk	↓Expression in cancer in 119 paired sample dataset.	Hong et al.^4^
Breast	↓Risk	↓Expression in cancer in 43 paired sample dataset. Associated with ↓ relapse free survival.	Hong et al.^4^
NSCLC	↓Risk	↓Expression in cancer in 20 paired sample dataset. Associated with ↓ relapse free survival.	Hong et al.^4^
HCC	↓Risk	↓Expression in cancer in 231 paired sample dataset.	Hong et al.^4^
CRC	↓Risk	↓Expression in cancer in 32 paired sample dataset.	Hong et al.^4^
Ovarian	↓Risk	↓Expression in ovarian data in The cancer genome atlas database.	Yang et al.^49^
RCC	↓Risk	↓mRNA Levels in RCC Negative Correlation with RCC Grade	Martuszewska et al.^27^
PDAC	↑Risk	↑Expression in 33 human PDAC samples.	Cheng et al.^50^
PDAC	↑Risk	↑Expression in PDAC cases. Utilized database containing 8280 cases and 6728 controls.	Zhu et al.^51^
GIST	↔Risk	Found that GIST samples had high degree of expression. Potential use in diagnostics. However, no observed change in patient outcome.	Salmikangas et al.^52^

In one notable exception, Cheng et al. (2018) demonstrated that *TNS2* and a receptor tyrosine kinase, AXL, were simultaneously upregulated in pancreatic ductal adenocarcinoma (PDAC) following an analysis of 33 human tissue samples and corroborative in vitro evidence (Table.2).^59^ Furthermore, Zhu et al. (2020) observed a similar relationship between *TNS2* expression and PDAC; in a large‐scale study containing 8280 PDAC cases and 6728 controls (Table.2).^60^ However, it is important to consider that neither of the pancreatic studies assessed for changes in overall survival or prognosis. Interestingly, these data would postulate a tumour‐specific pro‐oncogenic role for Tensin 2. Further examination of the molecular relationship between Tensin 2 and AXL is therefore warranted in pancreatic disease, especially given the clinical relevance and success of therapeutic tyrosine kinase inhibitors, and the desperate need for new therapeutic options in this field.

Salmikangas et al. (2022) demonstrated that relative to other sarcoma and gastrointestinal primaries, gastrointestinal stromal tumours (GIST) demonstrated a high level of TNS2 expression. However, there was no observed correlation between TNS2 expression and metastatic free/overall survival. It was noted the metastatic cases demonstrated lower expression of TNS2 compared to non‐metastatic samples; however, fewer metastatic cases were involved in the study (17/148 patients) (Table.2).^61^

### 8.3 Tensin 3

The clinical literature regarding Tensin 3 and cancer is again mixed. Early studies found *TNS3* expression to be negatively correlated in functional and non‐functional thyroid neoplasms (Table.3).^38^ Similarly, Martuszewska et al. (2009) observed decreased levels of mRNA in all Tensin proteins in renal cell carcinoma (RCC) and a negative correlation with RCC grade. Additionally, increased plasma membrane expression of Tensin 3 was correlated with better survival (Table.3).^15^ In contrast, a study by Chuang et al. assessing genomic signatures in acute myeloid leukaemia (AML) found *TNS3* expression to be significantly elevated in poor chemotherapy treatment responders.^62^ Furthermore, *TNS3* expression was correlated with decreased overall survival with a hazard ratio of 2.11 for overexpression, the 2nd highest risk of all genes studied (Table.3).^62^ These findings were further reflected in a separate study assessing pancreatic cancer by Klein et al. (2018) whereby a meta‐analysis of genome wide association studies (GWAS) of three international PDAC databases was conducted.^63^ From the bioinformatic search, a significant association between PDAC and a single nucleotide polymorphism (SNP) in the *TNS3* gene was identified (Table.3).^63^ Unfortunately, there was insignificant data to be able to interpret this finding in the context of overall survival, prognosis or clinical significance. However, due to the scale of the study, this finding certainly warrants further investigation.

**TABLE. 3:** Clinical data concerning Tensin3/TNS3 and effect on various cancer primaries. Renal Cell Carcinoma (RCC), Acute Myeloid Leukaemia (AML), Pancreatic Ductal Adenocarcinoma (PDAC).

Tensin 3/TNS3			
Primary	Effect on Cancer	Findings	Reference
Thyroid	↓Risk	↓Expression in functional + non‐functional Neoplasms.	Maeda et al.^22^
RCC	↓Risk	↓Expression in 233 RCC vs 48 normal human samples. If ↑expression in plasma membrane = ↑overall survival.	Martuszewska et al.^27^
AML	↑Risk	↑Expression in poor chemo response group, 104 patient sample. ↓Overall survival, hazard ratio of 2.11.	Chuang et al.^53^
PDAC	↑Risk	SNP in TNS3 gene association in PDAC. Data obtained in pooled PDAC GWAS dataset of 9040 cases and 12,496 controls.	Klein et al.^54^

## 9 DISCUSSION

Considering both the localization of Tensins to focal adhesions as well as their constituent binding sites to both integrins (via PTB domain) and multiple intracellular signalling proteins (via SH2 domain), it is logical that Tensin have been shown to have an important role in the tumour phenotype. The studies presented in this review highlight the important but often tumour‐specific role that Tensins 1–3 play in oncogenesis. We have segregated studies under the banner of selected Hanahan & Weinberg hallmarks, which demonstrates the impact that Tensins have on multiple biological pathways, with proliferative signalling, cell invasion and angiogenesis appearing prominent. Relationships and interplay between these are likely complex and indeed the inconsistencies and sometimes opposing effects suggest that further research on molecular mechanisms should focus on an organ/system‐based approach. We have suggested potential areas of study where appropriate.

However, an interaction between Tensin 1–3 and the tumour suppressor DLC1 was commonly observed (Figure 4, 5, 6) and may be particularly relevant, especially with recent studies of localization of DLC1 to focal adhesions.^21^, ^29^, ^64^, ^65^ This interaction appears to relate to the final effect of Tensin expression in a pro‐ or anti‐oncogenic manner, although sequencing studies of cancer cohorts fail to demonstrate DLC1 as an ubiquitous target for deletion in malignancy.^66^ Moreover, Tensin 1 and 3 in particular pose conflicting roles in tumour behaviour that cannot be explained by this single molecular interaction. Although there are some unique functions between the first three Tensin family members, that is F‐actin barbed end capping function in Tensin 1, it is likely that further inside‐out and outside‐in signal transduction pathways are relevant.^67^ Although we present simplified pathways of published evidence (Figures 1, 2, 3), large new data sequencing platforms will potentially aid in elucidating the associated pathways and likely provide more clinically relevant correlations and hypotheses to examine.

Indeed, we have attempted to summarize current clinical evidence for Tensin relevance in cancers. The conflicting role of Tensin 1 from in vitro studies is mirrored in clinical studies. This highlights the tumour‐specific nature of this protein's function, albeit its action in colorectal cancer appears to offer a convincing tumour promoting role (Table.1). Interestingly, both *Src* family kinases (SFKs) and DLC1 appear to play an important role in the pathogenesis of colorectal cancer.^68^, ^69^ Tensin 2 appears to have more consistent evidence for a tumour suppressive function. Despite this, a Tensin 2 variant lacking the C1 domain displays pro‐oncogenic behaviour as illustrated in Yam et al HCC study.^31^ Furthermore, the *TNS2* gene has been reported to be upregulated in PDAC genomic datasets. One theory for Tensin 2 contrasting function in pancreatic cancer may be due to a DLC1 deficiency in PDAC cells, similar to the dual behaviours observed in Tensin 3. Alternatively, Cheng et al. and Zhu et al. could both have observed a C1‐deficient Tensin 2 variant, the same protein responsible for Yam et al. more aggressive HCC phenotype.

Initially, the discovery of the Tensin3‐CTEN switch sparked significant interest and suggested Tensin 3 acted as a tumour suppressor with a clearly defined mechanism. Further studies corroborated the proposed anti‐migratory behaviour and linked the switch to DLC1 activity (Figure 6). However, these findings were observed in non‐neoplastic breast cell lines and broadening this work started to reveal pro‐neoplastic functions in certain cancer cell lines. As current clinical data are also unclear as to whether Tensin 3 acts to promote or supress neoplastic growth in a variety of tissue types, Src activity may aid in providing an explanation, given the previously explained potential interaction. This relationship between Tensin 3, DLC1 and Src is certainly a notable interaction worthy of further investigation.

Tensin biology is complex; the evidence presented helps focus discussion on relevant tumour types, interactions of interest and conflicting data. We await big data corroboration to help focus further research study. Localization to focal adhesions and potential interactions with signal transduction mechanisms make therapeutic targeting a distinct possibility. However, given the diverse potential effects, more robust evidence for a tumour‐specific, pro‐oncogenic effect is required.

## AUTHOR CONTRIBUTIONS

**Laurensius Mainsiouw:** Methodology (lead); writing – original draft (lead); writing – review and editing (lead). **Matthew Edward Ryan:** Supervision (supporting); writing – original draft (lead); writing – review and editing (lead). **Sassan Hafizi:** Writing – review and editing (supporting). **Jason Fleming:** Conceptualization (lead); supervision (lead); writing – review and editing (lead).

## CONFLICT OF INTEREST STATEMENT

The authors confirm that there are no conflicts of interest.

## Data Availability

Data sharing not applicable to this article as no datasets were generated or analysed during the current study.
